# 2652. Detection of respiratory viruses by air sampling and student and staff surveillance testing in two elementary schools

**DOI:** 10.1093/ofid/ofad500.2263

**Published:** 2023-11-27

**Authors:** Luke C Gard, Jennifer Goldman, Rangaraj Selvarangan, Anjana Sasidharan, Dithi Banerjee, Amanda M Hayes, Sydnie Petty, Olivia Almendares, Sadia Sleweon, Hannah L Kirking, Jennifer E Schuster

**Affiliations:** Children's Mercy Hospital-Kansas City, Kansas City, Missouri; Children's Mercy Hospital, Kansas City, Missouri; Children’s Mercy Kansas City, Kansas City, Missouri; Childrens Mercy Hospital, Missouri, Kansas; Children's Mercy Hospital, Kansas City, Missouri; Children's Mercy Hospital, Kansas City, Missouri; MCC-Longview, Raytown, Missouri; Centers for Disease Control and Prevention, Atlanta, Georgia; Center for Disease Control and Prevention, Atlanta, Georgia; Division of Viral Diseases, National Center for Immunization and Respiratory Diseases, CDC, Atlanta, Georgia; Children’s Mercy Kansas City, Kansas City, Missouri

## Abstract

**Background:**

The COVID-19 pandemic highlighted the need for better data to understand how ventilation may impact respiratory virus transmission. We evaluated if there was an association between respiratory viruses detected in school room air samples and nasal swabs from students/ staff in the same room.

**Methods:**

We performed air and respiratory sampling to detect respiratory viruses at 3 time points between Feb 27 - Mar 28, 2023 in 2 elementary schools. Three classrooms, a multipurpose room, and school nurse’s office in each school had 1 air sample/ room collected using Thermo Scientific AerosolSense™ sampler during 8 school hours. Concomitant respiratory viral testing, using self-collected nasal swabs, was performed in students/ staff enrolled in a respiratory virus surveillance school testing program. Air and respiratory specimens were tested for adenovirus (AdV); coronaviruses (CoV) 229E, HKU1, NL63, OC43; human metapneumovirus (hMPV), influenza A and B (Flu), parainfluenza virus 1-4 (PIV), respiratory syncytial virus (RSV), rhinovirus/ enterovirus (RV/EV), and SARS-CoV-2 using the Hologic® Panther Fusion® Respiratory Assay. Detected respiratory viruses in room air and respiratory samples were compared to determine likelihood of capturing current human respiratory viral infections in room air and additional viruses circulating from unsampled students/ staff in the same room. RV and AdV in human respiratory samples were typed by partial genomic sequencing to understand if viruses were similar.

**Results:**

Ten rooms, including 6 classrooms with ≈25 people/ room, were sampled at 3 occasions. At least 1 respiratory virus was detected in 27/ 30 (90%) air samples. 59 samples were obtained from 20 students and 5 staff in these rooms; 24 (41%) were positive for ≥1 virus and 11/24 (46%) rooms with concomitant air and respiratory samples had the same viruses detected (Table). RV typing demonstrates that both participants from Room E, Round 1 and 2 had RV-B27. Both participants from Room F, Round 2 with single AdV infections had AdV B3.

**Table.**
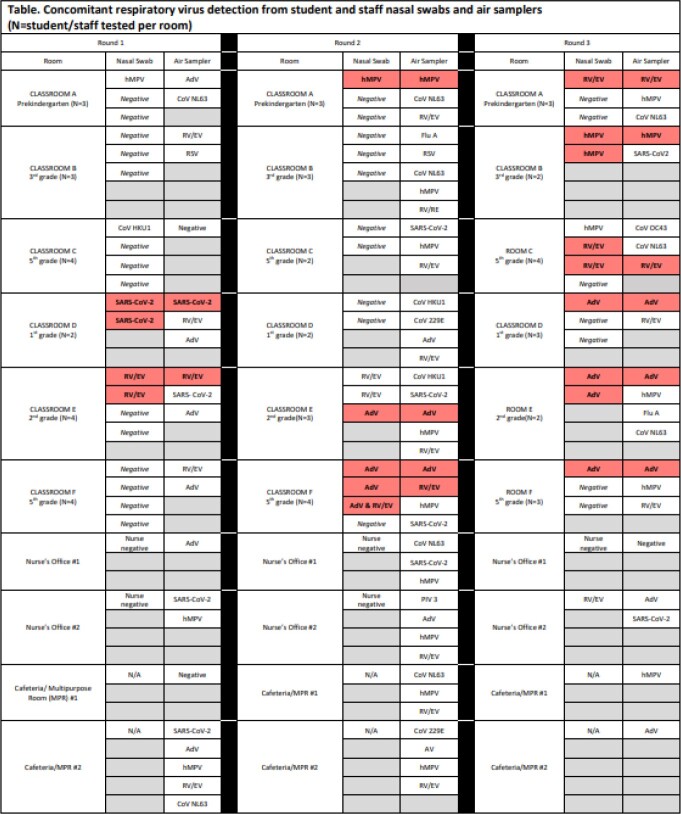
Concomitant respiratory virus detection from student and staff nasal swabs and air samplers (N=student/staff tested per room)

**Conclusion:**

Concomitant respiratory virus surveillance in air samples and students/ staff can be performed to better understand the role of ventilation and respiratory virus transmission. Additional studies, including sequencing data and symptom assessment, are needed.

**Disclosures:**

**Rangaraj Selvarangan, BVSc, PhD, D(ABMM), FIDSA, FAAM**, Abbott: Honoraria|Altona Diagnostics: Grant/Research Support|Baebies Inc: Advisor/Consultant|BioMerieux: Advisor/Consultant|BioMerieux: Grant/Research Support|Bio-Rad: Grant/Research Support|Cepheid: Grant/Research Support|GSK: Advisor/Consultant|Hologic: Grant/Research Support|Lab Simply: Advisor/Consultant|Luminex: Grant/Research Support

